# Diverse Coordinative Zinc Complexes Containing Amido-Pyridinate Ligands: Structural and Catalytic Studies

**DOI:** 10.3389/fchem.2018.00615

**Published:** 2019-01-04

**Authors:** Ming-Tsz Chen, Yu-Yang Chen, Guan-Lin Li, Chi-Tien Chen

**Affiliations:** ^1^Department of Applied Chemistry, Providence University, Taichung, Taiwan; ^2^Department of Chemistry, National Chung Hsing University, Taichung, Taiwan

**Keywords:** amidopyridinate ligands, amination reaction, zinc complexes, ring opening polymerization, biodegradable polymers, biocompatibility polymers

## Abstract

In this work, zinc complexes containing amidopyridinate ligands substituted with different pendant arms have been described. Treatment of ligand precursors with ZnEt_2_ at a 1:1 ratio in THF yields zinc ethyl complexes (PyN${\text{C}}_{1}^{\text{Py}}$)_2_(ZnEt)_2_ (**1**) and (PyN${\text{C}}_{2}^{\text{NMe}2}$)_2_(ZnEt)_2_ (**2**), respectively. Complexes **1** and **2** show the same geometry as a distorted tetrahedron, but adopt different coordination behaviors supported by the ligands. Complex **1** represents a rare and a non-centrosymmetric mode, which the amido group bridges two zinc centers to form a six-membered ring. However, complex **2** shows a centrosymmetric mode, which the pyridine group links to the zinc centers to form an eight-membered ring. Recrystallization of complex **2** gives an additional complex (PyN${\text{C}}_{2}^{\text{NMe}2}$)_4_Zn_3_(μ^3^-O) (**3**). We attempted to prepare zinc benzyl oxide complexes but afforded only a self-assembly cubane complex Zn_7_Et_6_(OBn)_8_ **(4)**. All molecular structures **1**–**4** are characterized depending on both single-crystal X-ray and spectroscopic data. Furthermore, their catalytic properties toward the ring opening polymerization of ε-caprolactone and L-lactide, using benzyl alcohol as the initiation reagent, are under investigation.

## Introduction

The role of polyesters has been given much attention due to their essential characteristics of being biodegradable, biocompatible, and permeable (Puelacher et al., 1994; Cheng et al., 1999). Owing to the promising applications of these polyesters, numerous groups have investigated and developed catalytic systems for generating polyesters by ring opening polymerization. Various kinds of metal complexes, such as Al, Mg, Zn, Fe, and Sn, have been well-studied as initiators/catalysts and control very well the molecular properties of the polymers (O'keefe et al., 2001; Coates, 2002; Nakano et al., 2003; Chisholm and Zhou, 2004; Coates and Moore, 2004; Wu et al., 2006; Wheaton et al., 2009). Low-toxic and environmentally friendly metal centers such as magnesium, calcium, and zinc are fascinating candidates with a crucial role in aiding to synthesize compounds with ancillary ligands (Wheaton et al., 2009). However, undesirable side reactions such as backbiting or transesterification often occur as a result of forming macrocycles over a wide range of molecular weight. Employing a steric bulky ligand or pendant arm(s) coordinated to an active metal center provides a barrier to hinder these undesired polymers. As a result of the successful application of β-diketiminate (BDI) complexes in ring opening polymerization, alternative or structurally similar ligand precursors are extensively studied. Recently, several systems have been checked for catalytic properties in ring opening polymerization (Cheng et al., 2000; Hill and Hitchcock, 2002; Gamer et al., 2005; Kröger et al., 2005, 2006; Lee et al., 2005; Vitanova et al., 2005; Alaaeddine et al., 2006; Chen et al., 2007, 2009, 2014; Liu et al., 2007; Wiecko et al., 2007; Gong and Ma, 2008; Yao et al., 2008; Peng and Chen, 2009; Chen and Chen, 2011; Peng et al., 2015). We are particularly motivated by the interest of the ligand precursors supported with parallel chelating systems and exhibited *iso*-electronic characteristics related to amidinato ligands. Our previous studies have focused on amidopyridine (Chen and Chen, 2017), and this article represents an extension of this work to investigate different substituent functional ligand precursors. According to our previous work on ring opening polymerization, the anilido-oxazolinate system (Chen et al., 2007, 2009; Chen and Chen, 2011), anilido-pyrazolate system (Peng and Chen, 2009), sulfon-amido-oxazolinate/pyrazolinate system (Chen et al., 2014), and the benzamidnated system (Wu et al., 2005) showed good activity; this is in addition to the β-diketiminate (BDI) zinc complex systems demonstrated by other groups (Cheng et al., 2000; Hill and Hitchcock, 2002; Gamer et al., 2005; Kröger et al., 2005, 2006; Lee et al., 2005; Vitanova et al., 2005; Alaaeddine et al., 2006; Chen et al., 2007, 2009, 2014; Liu et al., 2007; Wiecko et al., 2007; Gong and Ma, 2008; Yao et al., 2008; Peng and Chen, 2009; Chen and Chen, 2011; Peng et al., 2015). Hence, amidopyridinate zinc complexes are envisioned to be active players in ring opening polymerization. In support of this possibility, the preparation of zinc complexes supported with amido-pyridinate ligands has been investigated in this paper. Both coordination behaviors and catalytic activities in ring opening polymerization of L-lactide and ε-caprolactone by using benzyl alcohol as the initiation reagent are also under investigation.

## Materials and Methods

### Reagents and Methods

All manipulations were carried out under an atmosphere of dinitrogen using standard Schlenk-line or dry box techniques. Solvents were refluxed over the appropriate drying agent and distilled prior to use. DMSO (Dimethyl sulfoxide, TEDIA) was used as supplied. Deuterated solvents were dried over molecular sieves. ZnEt_2_ (Aldrich, 1.0M in hexane), CuI (Strem), K_3_PO_4_ (Lancaster), *L*-proline (Alfa), *N*,*N*-dimethylethyleneamine (Acros), and 2-(aminomethyl) pyridine (Acros) were used as supplied. Benzyl alcohol was dried over magnesium sulfate and distilled before use. ε-Caprolactone was dried over magnesium sulfate and L-Lactide was recrystallized from toluene prior to use.

^1^H and ^13^C{^1^H} NMR spectra were recorded either on Varian Mercury-400 (400 MHz) or Brucker-AV-400 (400 MHz) spectrometers in chloroform-*d* at ambient temperature unless stated otherwise and referenced internally to the residual solvent peak and reported as parts per million relative to tetramethylsilane. Elemental analyses were performed by Elementar Vario ELIV or FLASH 2000 Series Nitrogen and a Carbon Analyzer instrument (Thermo). The GPC measurements were performed in THF at 35°C with a Waters 1515 isocratic HPLC pump, a Waters 2414 refractive index detector, and Waters styragel column (HR4E). Molecular weights and molecular weight distributions were calculated using polystyrene as standard. The synthesis of the ligands and complexes is described in detail in the Supplementary Material in addition to the spectroscopic data. In a similar fashion, the details of the X-ray data can be found in the Supplementary Material.

### Ligand Synthesis

#### HNPy${\text{C}}_{1}^{\text{Py}}$(Foxon et al., 2002)

To a Schlenk flask containing 2-bromopyridine (0.48 mL, 5 mmol), 2-(aminomethyl) pyridine (0.77 mL, 7.5 mmol), K_3_PO_4_ (2.12 g, 10 mmol), CuI (0.1 g, 0.5 mmol), *L*-proline (0.12 g, 1 mmol), 3.5 mL DMSO was added at room temperature. The reaction mixture was heated to 110°C for 17 h. The resulting dark-brown solution was extracted with EA/H_2_O three times. The organic layer was dried over Na_2_SO_4_ and filtered. All volatiles were removed in vacuum to yield a yellow oily liquid product. Crude product was purified by flash column chromatography on silica gel (hexane/EA = 1:1 then methanol) to give a yellow liquid (The first band). Yield 1.36 g, 73%. ^1^H NMR (CDCl_3_, 400 MHz) δ 4.67(d, C*H*_2_, *J* = 5.6 Hz, 2H), 5.59(br, N*H*, 1H), 6.47(d, C*H-*Py, *J* = 8.4 Hz, 1H), 6.60(t, C*H-*Py, *J* = 6 Hz, 1H), 7.20(t, C*H-*Py, *J* = 6.2 Hz, 1H), 7.34(d, C*H-*Py, *J* = 7.6 Hz, 1H), 7.41(t, C*H-*Py, *J* = 6.8 Hz, 1H), 7.66(t, C*H-*Py, *J* = 7.6 Hz, 1Hz), 8.12(t, C*H-*Py, *J* = 4 Hz, 1H), 8.57(t, C*H-*Py, *J* = 4.8 Hz, 1H). ^13^C{^1^H} NMR (CDCl_3_, 100 MHz) δ 46.9(s, *C*H_2_), 107.4, 112.6, 121.3, 121.7, 136.3, 136.9, 147.7, 148.7(eight *C*H-Py), 158.1, 158.3(two tert-*C-*Py).

#### HNPy${\text{C}}_{2}^{\text{NMe}2}$ (Gogate et al., 2014)

The procedure for the preparation of HNPy${\text{C}}_{2}^{\text{NMe}2}$ was similar to that used for HNPy${\text{C}}_{1}^{\text{Py}}$ but with 2-bromopyridine (0.96 mL, 10 mmol), *N*,*N*-dimethylethyleneamine (3.28 mL, 30 mmol), K_3_PO_4_ (4.26 g, 20 mmol), CuI (0.19 g, 1 mmol), and *L*-proline (0.24 g, 2 mmol), and 8 mL DMSO. Crude product was purified by flash column chromatography on silica gel (hexane/EA = 1:1 then methanol) to give a yellow liquid (The first band). Yield 2.26 g, 91%. ^1^H NMR (CDCl_3_, 400 MHz) δ 2.26(s, N(C*H*_3_)_2_, 6H), 2.54(t, NC*H*_2_, *J* = 6Hz, 2H), 3.36(t, C*H*_2_NMe_2_, *J* = 5.6Hz, 2H), 5.00(s, N*H*, 1H), 6.40(d, C*H-*Py, *J* = 8.4 Hz, 1H), 6.54(t, C*H-*Py, *J* = 6.2 Hz, 1H), 7.40(t, C*H-*Py, *J* = 8.6 Hz, 1H), 8.08(d, C*H-*Py, *J* = 5.2 Hz, 1H). ^13^C{^1^H} NMR (CDCl_3_, 100 MHz) δ 39.3(s, *C*H_2_), 45.2(s, N(*C*H_3_)_2_), 58.0(s, *C*H_2_), 107.4, 112.5, 137.1, 148.1(four *C*H-Py), 158.8(one tert-*C-*Py).

### Synthesis of Zinc Complexes

#### (NPy${\text{C}}_{1}^{\text{Py}}$)_2_(ZnEt)_2_ (1)

To a flask containing HNPy${\text{C}}_{1}^{\text{Py}}$ (0.37 g, 2 mmol) and 15 mL hexane, 2.2 mL ZnEt_2_ (1M in hexanes, 2.2 mmol) was added at 0°C. The reaction mixture was allowed to warm to room temperature and reacted overnight. After 12 h of stirring, the yellow suspension solution was filtered and the residue was washed with hexane to afford a pale-yellow solid. Yield 0.38 g, 69%. ^1^H NMR (CDCl_3_, 400 MHz) δ −0.11(s, ZnC*H*_2_CH_3_, 2H), 0.88(m, ZnCH_2_C*H*_3_, 3H), 4.59(s, C*H*_2_, 2H), 6.31(t, C*H*-Py, *J* = 6 Hz, 1H), 6.61(d, C*H*-Py, *J* = 8.4 Hz, 1H), 7.16(m, C*H*-Py, 2H), 7.29(d, C*H*-Py, *J* = 8 Hz, 1H), 7.69(t, C*H*-Py, *J* = 7.6 Hz, 1H), 7.95(d, C*H*-Py, *J* = 4.4 Hz, 1H), 8.13(d, C*H*-Py, *J* = 4.4 Hz, 1H). ^13^C{^1^H} NMR(CDCl_3_, 100 MHz) δ −2.0(s, Zn*C*H_2_CH_3_), 13.0(s, ZnCH_2_*C*H_3_), 52.7(s, *C*H_2_), 109.4, 112.1, 122.2, 122.5, 136.5, 137.9, 146.6, 147.1(*C*H-Py), 161.4, 165.3(two tert-*C*-Py). Anal. Calc. for C_26_H_30_N_6_Zn_2_: C, 56.03; H, 5.43; N, 15.08. Found: C, 56.43; H, 5.19; N, 15.12.

#### (NPy${\text{C}}_{2}^{\text{NMe}2}$)_2_(ZnEt)_2_ (2)

To a flask containing HNPy${\text{C}}_{2}^{\text{NMe}2}$ (0.58 g, 3.53 mmol) and 15 mL THF, 3.89 mL ZnEt_2_ (1M in hexanes, 3.89 mmol) was added at 0°C. The reaction mixture was allowed to warm to room temperature and reacted overnight. After 12 h of stirring, the yellow solution was pumped and then washed with hexane to afford a yellow solid. Yield 0.81 g, 89%. ^1^H NMR (CDCl_3_, 400 MHz) δ 0.44(q, ZnC*H*_2_CH_3_, *J* = 7.6 Hz, 2H), 1.09(t, ZnCH_2_C*H*_3_, *J* = 8 Hz, 3H), 2.09(s, N(C*H*_3_)_2_, 6H), 2.48(t, C*H*_2_, *J* = 5.4 Hz, 2H), 3.26(br, C*H*_2_, 2H), 6.22(t, C*H*-Py, *J* = 8.2 Hz, 1H), 6.45(d, C*H*-Py, *J* = 8.8 Hz, 1H), 7.26(t, C*H*-Py, *J* = 7.8 Hz, 1H), 7.72(d, C*H*-Py, *J* = 4.4 Hz, 1H). ^13^C{^1^H} NMR(CDCl_3_, 100 MHz) δ −2.5(s, Zn*C*H_2_CH_3_), 13.3(s, ZnCH_2_*C*H_3_), 43.8(s, *C*H_2_), 45.9(s, N(*C*H_3_)_2_), 61.0(s, *C*H_2_), 107.5, 109.7, 137.1, 147.3(four *C*H-Py), 165.2(one tert-*C*-Py). Anal. Calc. for C_22_H_38_N_6_Zn_2_: C, 51.07; H, 7.40; N, 16.24. Found: C, 50.73; H, 7.50; N, 16.13.

#### Zn_7_Et_6_(OBn)_8_ (4)

Mostly, zinc benzyl oxide complexes not only served as better nucleophilic reagents but also as more effective catalysts for ROP reaction than zinc alkyl complexes. In our case, synthesizing zinc benzyl oxide complexes *via* general routes was unsuccessful, as only the self-assembly bis(cubane) zinc benzyl oxide complex **4** without the supporting ligand(s) was obtained. To a flask containing ligand precursors such as HNPy${\text{C}}_{1}^{\text{Py}}$ or HNPy${\text{C}}_{2}^{\text{NMe}2}$ and 15 mL THF, 1 equivalent ZnEt_2_ (1M in hexanes) was added at 0°C. The reaction mixture was allowed to warm to room temperature and reacted for 3 h. After 3 h of stirring, 1 equivalent benzyl alcohol was added and reacted for 3 h. Volatile compounds were removed under vacuum to afford a yellow solid. A white crystalline solid complex **4** (Zn_7_Et_6_(OBn)_8_) and some unknown compounds were obtained after the crystallization process. For an alternative method, a flask containing benzyl alcohol (0.71 mL, 6.84 mmol) and 15 mL hexane with 6 mL ZnEt_2_ (1M in hexanes, 6 mmol) was added at 0°C. The reaction mixture was allowed to warm to room temperature and reacted for 3 h. After filtration, a white solid was obtained. Yield 1.3 g, 96%. ^1^H NMR (CDCl_3_, 400 MHz) δ −0.32(m, ZnC*H*_2_CH_3_, 8H), −0.04(m, ZnC*H*_2_CH_3_, 4H), 0.72(m, ZnCH_2_C*H*_3_, 12H), 0.99(m, ZnCH_2_C*H*_3_, 6H), 4.76(s, C*H*_2_Ph, 4H), 4.92(s, C*H*_2_Ph, 4H), 5.04(s, C*H*_2_Ph, 8H), 7.26(br s, C*H*-Ph, 3H), 7.38(m, C*H-*Ph, 28H), 7.49(br d, C*H*-Ph, *J* = 8.2 Hz, 9H). ^13^C{^1^H} NMR(CDCl_3_, 100 MHz) δ −1.8(s, Zn*C*H_2_CH_3_), 0.1(s, ZnCH_2_*C*H_3_), 12.1(s, ZnCH_2_*C*H_3_), 12.5(s, ZnCH_2_*C*H_3_), 69.0(s, O*C*H_2_Ph), 70.1(s, O*C*H_2_Ph), 70.9(s, O*C*H_2_Ph), 127.7(s, *C*H-Ph), 128.1(d, *C*H-Ph), 128.6(s, *C*H-Ph), 128.9(d, *C*H-Ph), 129.8(s, *C*H-Ph), 140.7, 141.5(two tert-*C*-Ph). Anal. Calc. for C_68_H_86_O_8_Zn_7_: C, 54.84; H, 5.82. Found: C, 54.35; H, 5.25.

### X-Ray Data Collection and Structure Refinement

Crystals **1**–**4** were grown from concentrated THF or hexane solution and isolated by filtration. The crystal was mounted onto a cryoloop and transferred into a cold nitrogen gas stream. Data were collected by a mounted Bruker AXS SMART 1000 diffractometer with graphite-monochromated Mo-Kα radiation (λ = 0.7107 Å). Absorption correction was applied using SADABS (Sheldrick, 1996, 1997). Unit-cell parameter refinement, integration, and data reduction were carried out with the SAINT program (Brucker). The structure was solved by direct methods using a SHELXTL package (Spek, 2003, 2015; Sheldrick, 2015). All non-H atoms were located from successive Fourier maps, and hydrogen atoms were refined using a riding model. Anisotropic thermal parameters were used for all non-H atoms, and fixed isotropic parameters were used for H atoms.

CCDC 1863455–1863458 were contained in the Supplementary Material for this paper. The detailed crystal data can be obtained free of charge from The Cambridge Crystallographic Data Centre at the following web address: www.ccdc.cam.ac.uk/data_request/cif.

### General Procedure for the Ring Opening Polymerization Reaction

To a flask containing the prescribed amount of monomer (L-lactide or ε-caprolactone) and catalyst (0.05 mmol for L-lactide; 0.125 mmol for ε-caprolactone), 10 mL (for L-lactide) or 15 mL (for ε-caprolactone) of solvent were added. The reaction mixture was stirred at the prescribed temperature for the prescribed time. After the reaction was quenched by the addition of 10 mL acetic acid solution (0.35 N), the resulting mixture was poured into 50 mL *n*-heptane to precipitate polymers. Crude products were recrystallized from THF–hexane and dried *in vacuo* up to a constant weight.

## Results and Discussion

**Figure 1:**
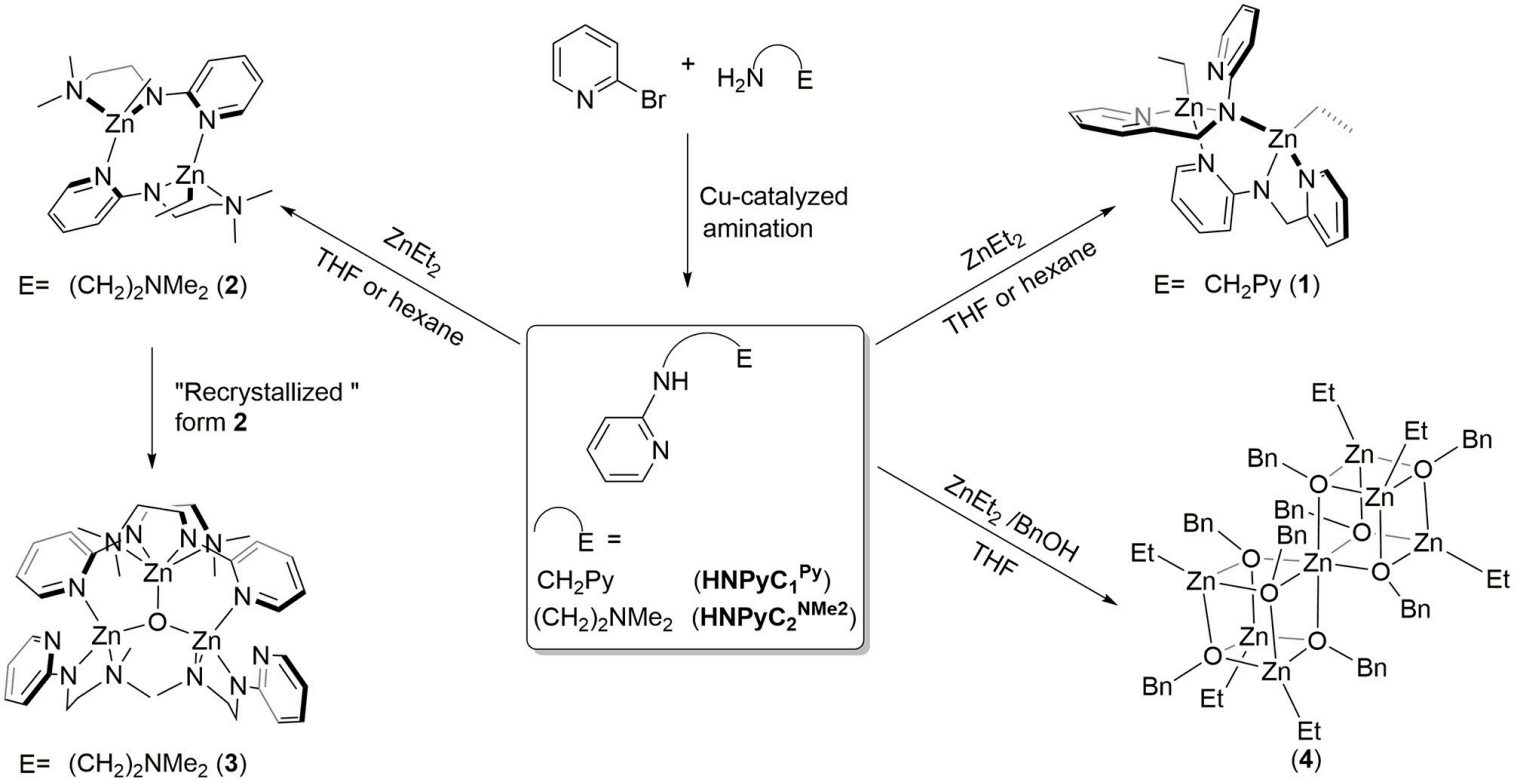
Synthesis of amido-pyridinate zinc complexes.

The ligand precursors HNPy${\text{C}}_{\text{n}}^{\text{E}}$ (*n* = 1, E = Py; *n* = 2, E = NMe_2_) with aliphatic pendant functionalities were prepared from the copper(I)-catalyzed amination (Zhang et al., 2005) of 2-bromopyridine with suitable amines (2-aminomethyl pyridine for HNPy${\text{C}}_{1}^{\text{Py}}$; *N*,*N'*-dimethylethylene diamine for NHPy${\text{C}}_{2}^{\text{NMe}2}$) containing CuI, *L*-proline and K_3_PO_4_ in DMSO at 110°C for 17 h to give moderate yields (73% for HNPy${\text{C}}_{1}^{\text{Py}}$; 91% for NHPy${\text{C}}_{2}^{\text{NMe}2}$), following previously-established procedure (Chen et al., 2007, 2009; Chen and Chen, 2011). The ligand precursors, HNPy${\text{C}}_{\text{n}}^{\text{E}}$ are more easily synthesized by copper catalysis under air-controlled conditions than with other preparations (Foxon et al., 2002; Liu et al., 2007). In Figure 1, detailed synthetic routes and the depicted structures are introduced. In order to explore the potential catalytic properties of amidopyridinate zinc complexes, zinc complexes have been synthesized using HNPy${\text{C}}_{\text{n}}^{\text{E}}$ with ZnEt_2_ in a 1:1 molar ratio in THF or hexane isolating in the generation of zinc ethyl complexes **1–2**. Complex **1** adopts mismatched coordination behavior in supporting the ligand, but complex **2** represents a centrosymmetric modular coordination system with the ligand. After purifying complex **2** with hexane, the filtrate is recrystallized to give zinc oxide complex **3**. However, further data could not be obtained from the methodology suggested in the literature (Murso and Stalke, 2004), where the reaction was carried out by the treatment of ligand precursor NHPy${\text{C}}_{2}^{\text{NMe}2}$ with an equal equivalent of ^n^BuLi followed by the addition of ZnCl_2_. This was also true when an equivalent of water reacted with ZnEt_2_ and the ligand precursor also failed to get complex **3**. In general, zinc benzyl oxide complexes exhibited better nucleophilic properties than zinc alkyl complexes, thus should be effective catalysts for ROP reaction (O'keefe et al., 2001). Attempts to synthesize zinc benzyl oxide complexes proven to be unsuccessful, with only the self-assembly bis(cubane) zinc benzyl oxide complex **4** without the supporting ligand(s) being obtained. Alternatively, complex **4** can also be introduced directly by using ZnEt_2_ with one equivalent benzyl alcohol in hexane. The spectroscopic analysis data of **1–2** and **4** correspond to the structures depicted in Figure 1. All of them are fully identified not only with NMR spectroscopy but also with elemental analyses.

**Figure 2:**
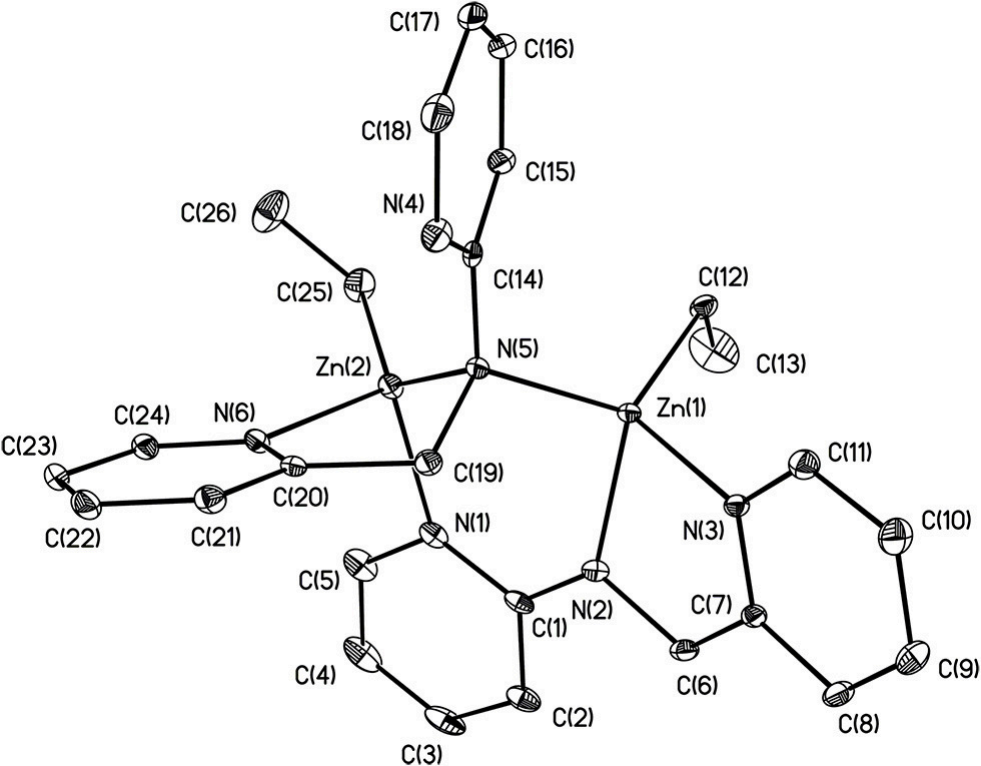
Molecular structure of **1**. Selected bond lengths (Å) and bond angles (°): Zn(1)-N(2), 2.023(2); Zn(1)-N(3), 2.115(2); Zn(1)-N(5), 2.078(2); Zn(1)-C(12), 1.996(3); Zn(2)-N(1), 2.090(2); Zn(2)-N(5), 2.088(2); Zn(2)-N(6), 2.136(2); Zn(2)-C(25), 1.985(3); C(1)-N(1), 1.372(4); C(1)-N(2), 1.330(3); C(1)-C(2), 1.435(4); C(14)-N(4), 1.335(4); C(14)-N(5), 1.399(3); N(2)-Zn(1)-N(3), 79.81(9); N(3)-Zn(1)-N(5), 106.37(8); C(12)-Zn(1)-N(2), 123.26(11); C(12)-Zn(1)-N(3); 117.04(10); N(1)-Zn(2)-N(5), 106.85(8); N(5)-Zn(2)-N(6), 80.89(8); C(25)-Zn(2)-N(5), 132.04(12); C(25)-Zn(2)-N(1), 111.13(12). Hydrogen atoms on carbon atoms omitted for clarity.

**Figure 3:**
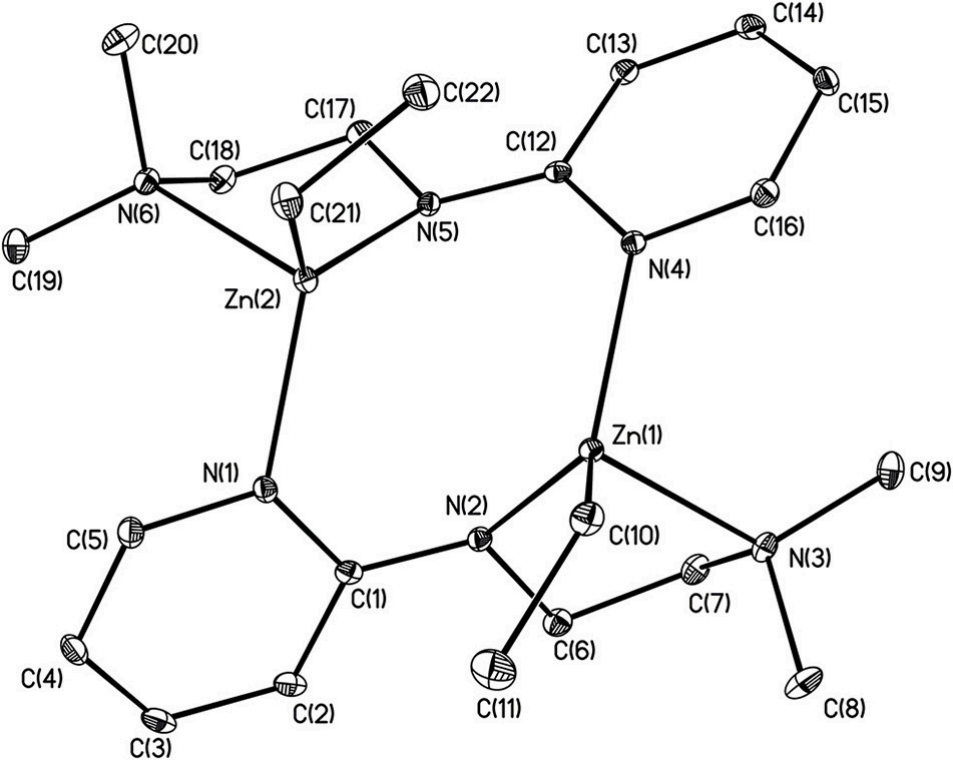
Molecular structure of **2**. Selected bond lengths (Å) and bond angles (°): Zn(1)-N(2), 2.0142(14); Zn(1)-N(3), 2.2424(14); Zn(1)-N(4), 2.0667(13); Zn(1)-C(10), 1.9915(16); Zn(2)-N(1), 2.0716(14); Zn(2)-N(5), 2.0133(13); Zn(2)-N(6), 2.2620(14); Zn(2)-C(21), 1.9928(17); C(1)-N(1), 1.370(2); C(1)-N(2), 1.339(2); C(1)-C(2), 1.430(2); C(12)-N(4), 1.372(2); C(12)-N(5), 1.333(2); N(2)-Zn(1)-N(4), 111.86(5); N(3)-Zn(1)-N(4), 98.43(15); C(10)-Zn(1)-N(3), 112.07(6); C(10)-Zn(1)-N(2); 122.67(6); N(1)-Zn(2)-N(5), 11 1.20(5); N(5)-Zn(2)-N(6), 81.67(5); C(21)-Zn(2)-N(5), 125.07(6); C(21)-Zn(2)-N(1), 118.10(6). Hydrogen atoms on carbon atoms omitted for clarity.

**Figure 4:**
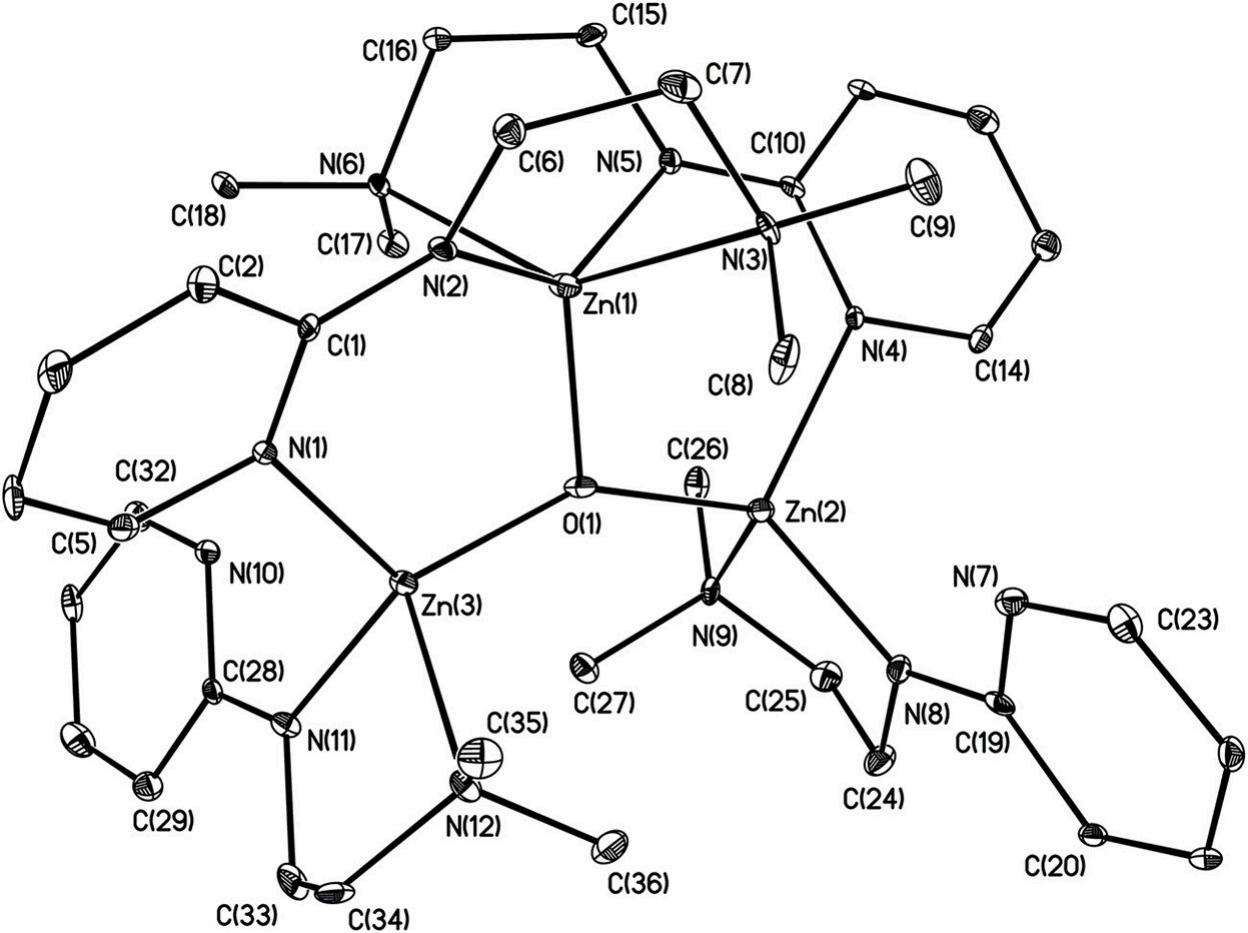
Molecular structure of **3**. Selected bond lengths (Å) and bond angles (°): Zn(1)-N(2), 1.998(3); Zn(1)-N(3), 2.458(3); Zn(1)-N(5), 2.001(3); Zn(1)-N(6), 2.397(3); Zn(1)-O(1), 1.833(2); Zn(2)-N(4), 2.070(3); Zn(2)-N(8), 1.956(3); Zn(2)-N(9), 2.192(3); Zn(2)-O(1), 1.864(2); Zn(3)-N(1), 2.087(3); Zn(3)-N(11), 1.935(3); Zn(3)-N(12), 2.195(3); Zn(3)-O(1), 1.864(2); Zn(1)-O(1)-Zn(2), 107.45(11); Zn(1)-O(1)-Zn(3), 107.36(12); N(3)-Zn(1)-N(2), 75.07(11); N(5)-Zn(1)-N(6); 75.91(10); N(8)-Zn(2)-N(9), 82.87(12); N(4)-Zn(2)-O(1), 112.01(11); N(1)-Zn(3)-O(1), 111.67(12); N(11)-Zn(3)-O(1), 133.86(12). Hydrogen atoms on carbon atoms omitted for clarity.

**Figure 5:**
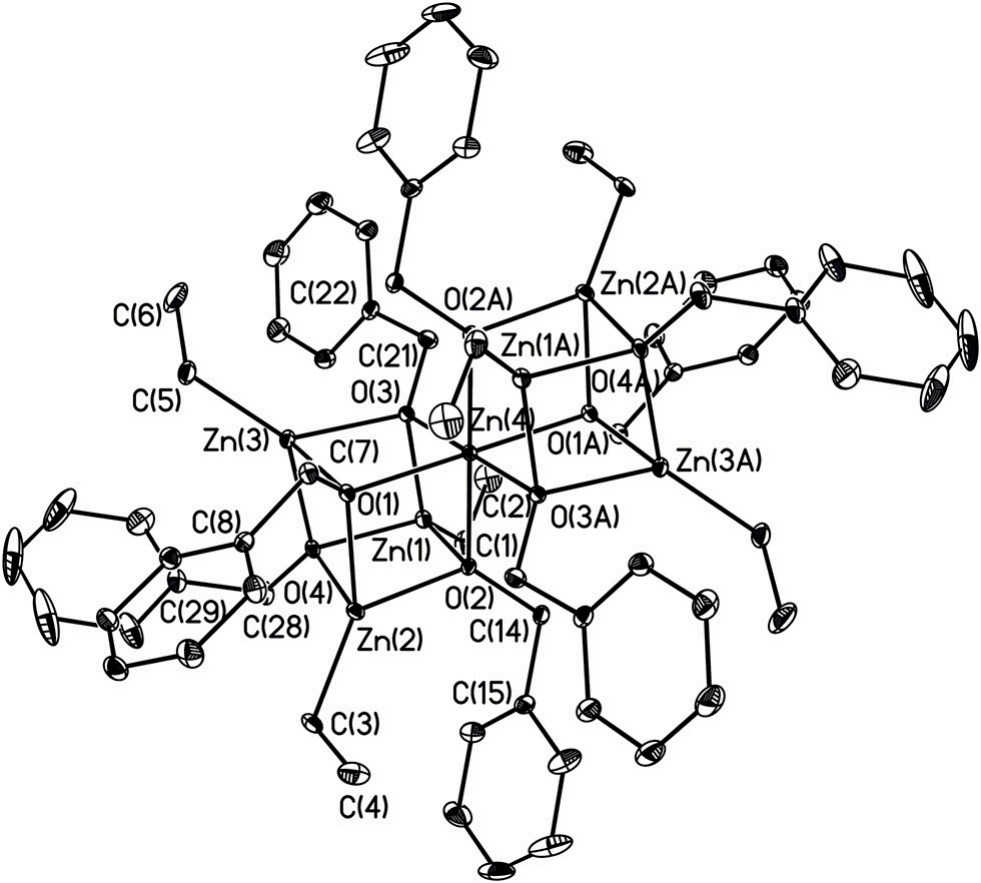
Molecular structure of **4**. Selected bond lengths (Å) and bond angles (°): Zn(1)-C(1), 1.963(3); Zn(1)-O(2), 2.060(2); Zn(1)-O(3), 2.054(2); Zn(1)-O(4), 2.055(2); Zn(2)-C(3), 1.962(3); Zn(2)-O(2), 2.090(2); Zn(2)-O(1), 2.060(2); Zn(2)-O(4), 1.968(3); Zn(3)-O(3), 2.084(2); Zn(3)-C(5), 1.968(3); Zn(4)-O(1), 2.111(2); Zn(4)-O(2), 2.108(2); Zn(4)-O(3), 2.131(2); O(3)-C(2), 1.412(4); C(21)-C(22), 1.501(4); O(4)-C(28), 1.443(4); C(28)-C(29), 1.495(5); C(1)-Zn(1)-O(3), 133.66(13); O(2)-Zn(1)-O(3), 85.87(8); O(2)-Zn(1)-O(4), 84.71(8); Zn(1)-O(3)-Zn(3), 95.96(8); Zn(4)-O(3)-Zn(3), 97.61(9); Zn(4)-O(3)-C(21), 118.15(18); Zn(4)-O(1)-C(7), 120.77(18); O(2)-Zn(2)-O(4), 82.34(8); O(2)-Zn(2)-C(3), 85.87(8); O(2)-Zn(2)-O(1), 83.12(8); C(28)-O(4)-Zn(4), 114.47(19). Hydrogen atoms on carbon atoms omitted for clarity.

X-ray structures **1–4** are determined and are obtained from concentrated hexane or concentrated THF solutions. All crystal structural images and data are summarized in Figures 2–5. The molecular structure of Complex **1** represents a non-centrosymmetric dimer formed by the association of two [NPy${\text{C}}_{1}^{\text{Py}}$]^−^ moieties, which exhibit a 6-membered ring core motif as a distorted chair form (Figure 2). Two four-coordinate Zn atoms featuring the terminally Zn-bound ethyl groups comprised complex **1**. The geometry of zinc center **1** can be seen as a distorted tetrahedral geometry and uses one amido nitrogen atom N(5) to bridge two zinc centers. This coordination behavior is limited and not easily found in the literature. Each zinc atom is four-coordinate, which exhibited the bonding mode comprising one nitrogen atom of the pyridyl groups with Zn(1)–N(3), Zn(2)-N(1), Zn(2)-N(6) bond distances of 2.115(2), 2.090(2), 2.136(2)Å and one nitrogen atom of the amido groups with Zn(1)–N(2), Zn(1)–N(5), Zn(2)–N(5) bond distances of 2.023(2), 2.078(2), 2.088(2)Å. Complex **2** exists as a centrosymmetric dimer and shows as an 8-membered ring core motif which is similar to zinc pyridyl systems (Zheng et al., 2008). The zinc centers have a distorted tetrahedral geometry. The metal centers of the zinc atoms are four-coordinate, which demonstrated the bonding mode, including one nitrogen atom of the amine groups with Zn(1)–N(3), Zn(2)–N(6) bond distances of 2.2424(14), 2.2620(14)Å, one nitrogen atom of the amido groups with Zn(1)–N(2), Zn(2)–N(5) bond distance of 2.0142(14), 2.0133(13)Å, and one nitrogen atom of the pyridyl groups with Zn(1)–N(4), Zn(2)–N(1) bond distance of 2.0677(13), 2.0716(14)Å. The solid state structure of **3** is comprised of three zinc cations, a single oxygen O^2−^-dianion and four monoanionic ligand fragments as shown in Figure 4. Two metal centers, Zn(2) and Zn(3), have a distorted tetrahedral geometry with one μ_3_-O atom and two nitrogen atoms. The other metal center [Zn(1)] shows a distorted square pyramidal geometry with one μ_3_-O atom and four nitrogen atoms. In complex **3**, the Zn–O bond distances are 1.883(3), 1.866(2) and 1.863(2)Å showing two different bonding lengths. Two different coordinate modes are demonstrated by four ligands, one type being the uncoordinated pyridyl group, and this could be caused by the balance and bond distances (Murso and Stalke, 2004). Figure 5 shows the solid state plot of **4**. There are two shared Zn-O cubes in this molecule. Two bonding modes for zinc centers in complex **4**, and six of the Zn atoms, are *T*_*d*_ geometry with the shared Zn adopting an *O*_*h*_ geometry. Adopting with four μ_3_-O_benzyl_ and three terminal ethyl groups fills the metal center geometry (for each individual cubane); for the Td bonding mode of Zn atoms three μ3-Obenzyl groups are used, whereas for the Oh-bound Zn atoms six μ3-Obenzyl groups are used. The Zn-O bonds to the central Zn(1) atom are approximately 0.077Å longer than those to the other Zn atoms. The bonding angles of Zn-O-Zn are generally larger [94.90(8)−97.69(9)°] than the bonding angles of O-Zn-O [81.47(8)−83.91(6)°]; the angles are within one cube and akin to the reported literature (Ishimori et al., 1976; Lewinski et al., 2003, 2008; Boyle et al., 2004; Jana et al., 2007; Tsaroucha et al., 2011; Prochowicz et al., 2014).

**Figure 6:**
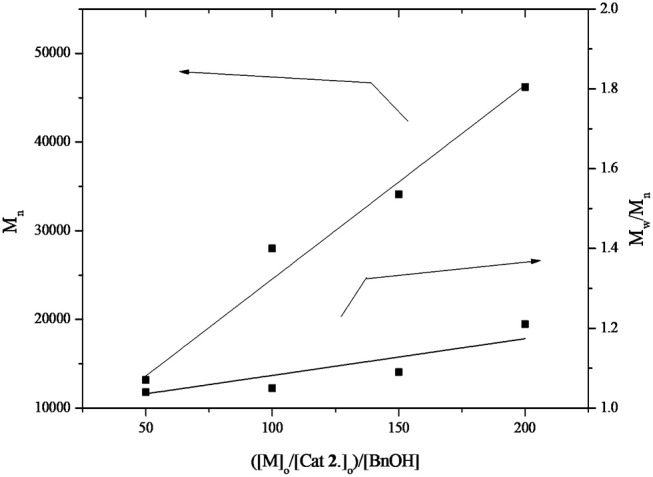
Polymerization of L-lactide catalyzed by **2** in toluene at 50°C.

**Figure 7:**
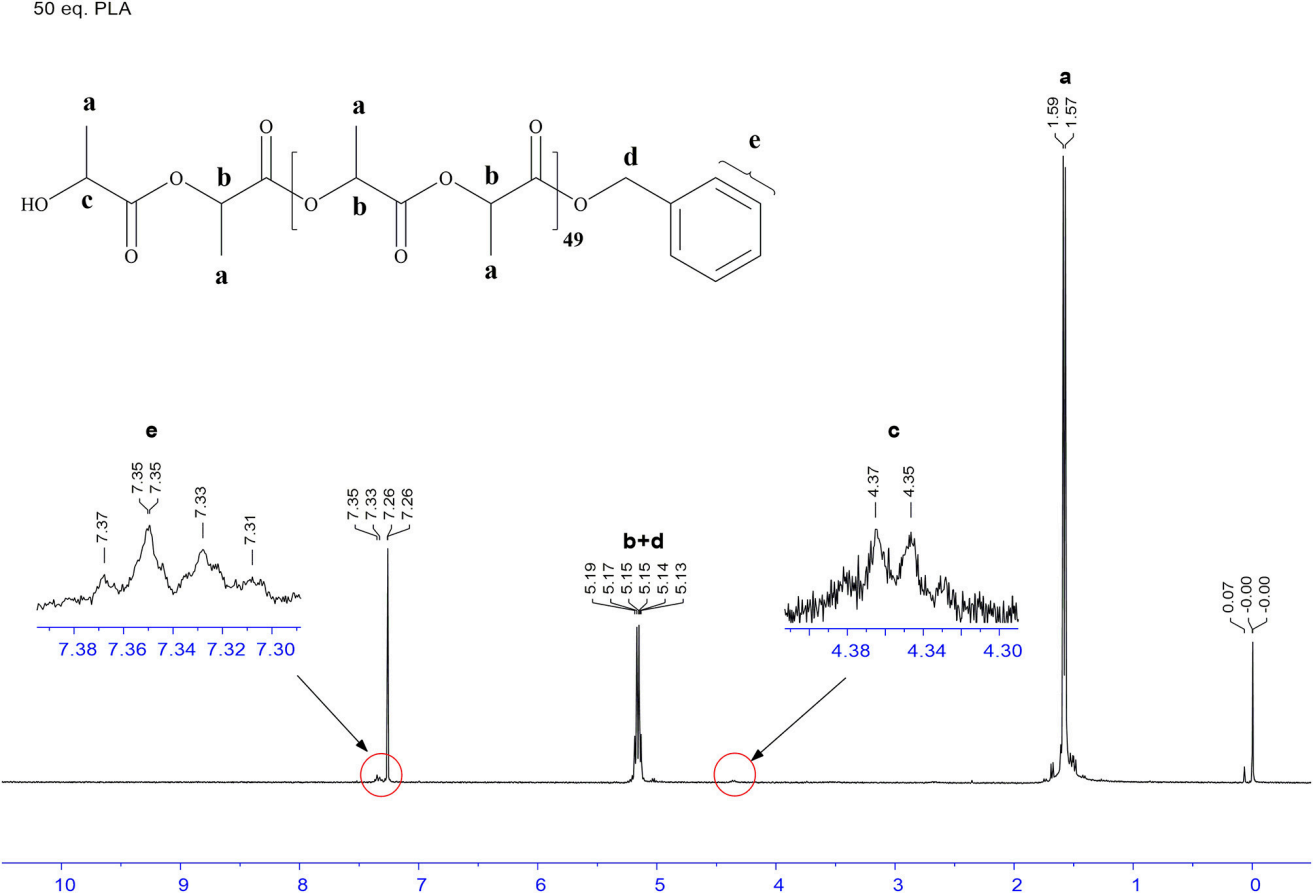
^1^H NMR spectrum of PLLA-50 catalyzed by **2** in toluene at 50°C.

**Figure 8:**
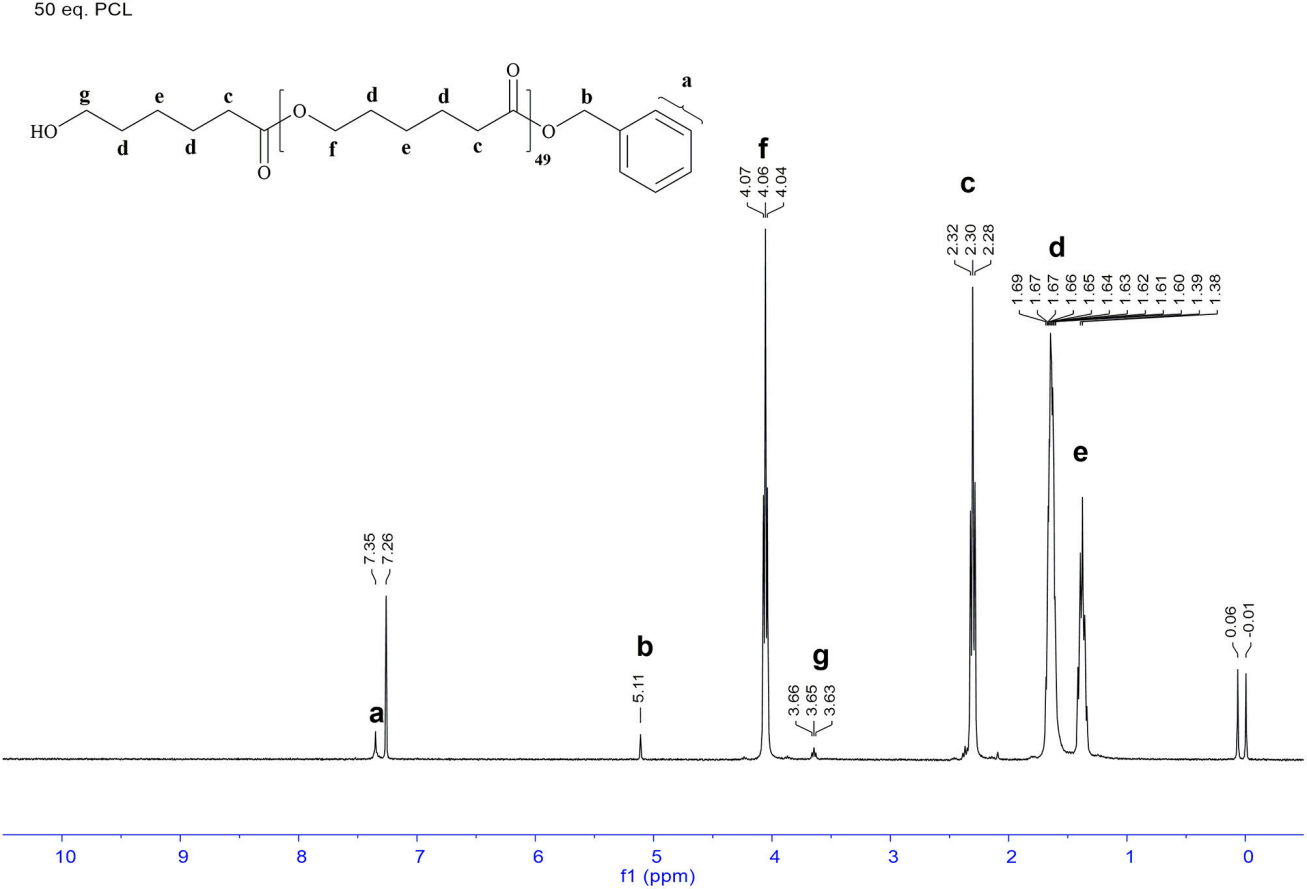
^1^H NMR spectrum of PCL-50 catalyzed by 2 in toluene at 50°C.

Undoubtedly, BDI zinc complexes are very active catalytic species/systems in ring opening polymerization (ROP), but to our best knowledge, amidopyridinate complexes provide limited information toward this aspect. Based on the previous work on anilido-pyridinate zinc system for ROP (Chen and Chen, 2017), the optimized solvent/temperature combination is revealed to be 10 mL toluene at 50°C, using benzyl alcohol as the transfer/initiation reagent after several trials on polymerization with CH_2_Cl_2_, THF and toluene. The same conditions were used to check the activities of the three catalysts **1**, **2**, and **4**. Polymerization of L-lactide, carried out by applying complexes **1, 2**, and **4** as catalysts by using benzyl alcohol as the initiation reagent, has been methodically studied and operated under a dry nitrogen atmosphere. In Table 1, representative data are summarized and listed. Notably, experimental results show that complexes **1** and **2** demonstrate more positive activities than **4** within the same period (entries 1–3), whereas **4** exhibits an inferior conversion at 50°C up to 120 min (entry 3). But when loading the high ratios of monomer at 50°C (entry 6), poor PDI is demonstrated by complex **1**, with the reasonable assumption that this is caused by the interactions between pendant functionality of ligand precursors, the metal center and the rigidity of pyridyl group, which impedes the attraction of benzyl alcohol or monomer to the metal center, resulting in a hinderance of the propagation process in the polymerization. Referred to the same monomer loading, complex **2** shows a more controlled manner (entry 10). In addition, the kinetic studies of complexes **1** and **2** for polymerization L-lactide were carried out with benzyl alcohol, and complex **1** held better activities than did complex **2** (see Supplementary Material). Because of the easy preparation and manipulable attributes, complex **2** was utilized for investigation in the presence of optimized conditions. The linear relationship between the number-average molecular weight (M_n_) and the monomer-to-initiator ratio ([M]_0_/[I]_0_) demonstrated in Figure 6 (entries 2, 7–10) implies the “living” character of the polymerization process. Analysis of the produced polymer end group is testified by the ^1^H NMR spectrum and the polymer sample is obtained from L-lactide and **2** ([M]_0_/[BnOH] = 50). All peaks are assignable and corresponded to the proposed structure capped with the benzyl alkoxyl group, as shown in Figure 7. This indicates the Zn complex/BnOH catalytic behavior might follow the pathway as an “activated monomer,” where it or BnOH are activated by complex **2** and BnOH plays as a nucleophilic role (Huang et al., 2008; Romain et al., 2017). Both complexes **1** and **2** show similar activities, but complex **2** with NMe_2_ substituted with functional group ligands exhibits more controllable behavior for polymerization. Complexes **1, 2**, and **4** were also studied to demonstrate their catalytic behavior in the ROP of ε-caprolactone (CL). Representative results are also summarized in Table 1 (entries 11–24). Selected experimental results indicate that complex **2** has a more easily controlled disposition for catalyzing ROP of CL compared to complexes **1** and **4** (entries 11–19). Complex **4** demonstrates improved activity for polymerization of CL compared to L-lactide (entry 22 vs. 3). The resulting polymers of PCLs give reasonable number-average molecular weight (M_n_) and poly dispersity index (PDI) values (1.08–1.27). The relationship of M_n_ vs. ([M]_0_/[I]_0_) is illustrated by the data conducted by complex **2**; it possesses a roughly linear relationship and carries out polymerization in a controlled manner. The ^1^H NMR analysis of the end group is also carried out and the result is similar to PLA (Figure 8). This result means that polymerization of ε-caprolactone followed the same catalytic behavior with L-lactide. Depending on catalytic results, we can conclude complexes **1** and **2** should proceed via a different pathway in the ROP reaction compared to complex **4**.

**Table 1:** Polymerization of L-Lactide and ε-caprolactone using complexes **1–2**, **4** at 50°C^a^.

**Entry**	**Monomer**	**Solvent**	**Cat**.	**{[M]_**0**_:[Zn]_**0**_}:[BnOH]**	**Time (min)**	**M_**n**_ (obsd)^**b**^**	**M_**n**_ (calcd)^**c**^**	**Conv.(%)^**d**^**	**Yield (%)^**e**^**	**M_**w**_/${\text{M}}_{\text{n}}^{\text{b}}$**
1	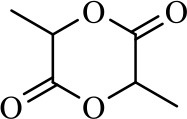	Toluene	**1**	50:1	15	10,600 (6,200)	6,900	94	79	1.16
2		Toluene	**2**	50:1	15	11,800 (6,800)	6,900	94	75	1.07
3		Toluene	**4**	50:1	120	–	–	15	–	–
4		Toluene	**4**	50:1	1,440	21,000 (12,200)	7,200	98	82	1.38
5		Toluene	**1**	200:1	45	40,300 (23,400)	28,400	98	86	1.35
6		Toluene	**1**	300:1	50	27,700 (16,100)	42,100	97	88	2.20
7		Toluene	**2**	100:1	15	28,000 (16,200)	13,500	93	82	1.05
8		Toluene	**2**	150:1	30	34,100 (19,800)	18,900	87	73	1.09
9		Toluene	**2**	200:1	45	46,200 (26,800)	27,200	94	84	1.21
10		Toluene	**2**	300:1	50	38,200 (22,200)	41,600	96	83	1.35
11^f^	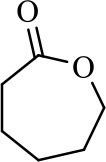	THF	**2**	50:1	120	6,600 (3,700)	5,200	90	78	1.21
12^f^		DCM	**2**	50:1	120	–	–	Trace	–	–
13^f^		Toluene	**2**	50:1	120	15,100 (8,500)	5,800	99	80	1.22
14^f^		THF	**1**	50:1	120	–	–	62	–	–
15^f^		DCM	**1**	50:1	120	–	–	13	–	–
16^f^		Toluene	**1**	50:1	120	14,100 (7,900)	5,500	95	80	1.35
17^f^		THF	**4**	50:1	120	–	–	60	–	–
18^f^		DCM	**4**	50:1	120	–	–	27	–	–
19^f^		Toluene	**4**	50:1	120	10,300 (5,800)	4,400	75	–	1.12
20		Toluene	**1**	50:1	8	11,100 (6,200)	4,900	84	72	1.14
21		Toluene	**2**	50:1	8	11,200 (6,300)	5,600	97	83	1.08
22		Toluene	**4**	50:1	60	4,900 (2,700)	5,800	99	78	1.16
23		Toluene	**2**	100:1	15	21,800 (12,200)	11,000	95	82	1.11
24		Toluene	**2**	150:1	20	32,300 (18,100)	16,900	98	87	1.27

a *For L-Lactide: 10 mL solvent, [Zn]_0_ = 0.005M, [BnOH] = 0.005M; for ε-caprolactone: 15 mL solvent. [Zn]_0_ = 8.33mM; [BnOH] = 8.33mM*.

b *Obtained from GPC analysis and calibrated by polystyrene standard. Values in parentheses are the values obtained from GPC times 0.58 for PLA; 0.56 for PCL*.

c *Calculated from [Mw(monomer) × [M]_0_/[Zn]_0_ × conversion /([BnOH]_(eq)_] + M(BnOH))*.

d *Obtained from ^1^H NMR analysis*.

e *Isolated yield*.

f *T = 26°C*.

## Conclusions

We have reported the preparation and full characterizations of new amidopyridinate zinc complexes. Two coordinate modes of zinc ethyl complexes supported with amidopyridinate ligands and one self-assembly zinc benzyl oxide cubane complex were created and investigated. Under optimized conditions, amidopyridinate zinc complexes demonstrate efficient activities for the ring opening polymerization of L-lactide and ε-caprolactone when employing benzyl alcohol as the initiation reagent. Initial studies are focusing on advancing the fine-tuning of ligand precursors and more advanced research will pay more attention to applying metal complexes for the undergoing catalytic reactions.

## Author Contributions

All authors listed have made a substantial, direct and intellectual contribution to the work, and approved it for publication.

### Conflict of Interest Statement

The authors declare that the research was conducted in the absence of any commercial or financial relationships that could be construed as a potential conflict of interest.
