# Supplementary material for: Diverse Coordinative Zinc Complexes Containing Amido-Pyridinate Ligands: Structural and Catalytic Studies
Source: Front Chem. 2019 Jan 4;6:615. doi: 10.3389/fchem.2018.00615 (PMC6328485; doi:10.3389/fchem.2018.00615)
Supplement: Supplementary file 1 [file Table_1.DOCX]

Supplementary Material

Diverse Coordinative Zinc Complexes Supporting with Amido-Pyridinate Ligands: Structural and Catalytic Studies

Ming-Tsz Chen^1*^, Yu-Yang Chen^1^, Guan-Lin Li ^1^, Chi-Tien Chen^2*^

*** Correspondence:** Corresponding Author: M.-T. Chen: [mschen@pu.edu.tw](mailto:mschen@pu.edu.tw); C.-T. Chen: [ctchen@dragon.nchu.edu.tw](mailto:ctchen@dragon.nchu.edu.tw)

*^1^ Department of Applied Chemistry, Providence University, Taichung 43301, Taiwan, ROC.*

^2^Department of Chemistry, National Chung Hsing University, Taichung 402, Taiwan, ROC.

**Table of Contents**

Spectroscopic data of ligand precursors…………………………………………...…………………S2

Spectroscopic data of complexes **1**, **2**, **4**…………………………………………...…………………S4

Crystallographic Information……………………………………….………………….……….….S7

Kinetic Studies for Polymerization of L-lactide by Complexes **1** and **2**………………………………S8

Spectroscopic data of **HNPyC_1_^Py^**

Spectroscopic data of **HNPyC_2_^NMe2^**

Spectroscopic data of complex **1**


Spectroscopic data of complex **2**


Spectroscopic data of complex **4**


**Table S1.** Summary of crystal data for complexes **1**-**4**.

|  | **1** | **2** | **3** | **4** |
| --- | --- | --- | --- | --- |
| Formula | C_26_H_30_N_6_Zn_2_ | C_22_H_38_N_6_Zn_2_ | C_36_H_56_N_12_OZn_3_ | C_68_H_86_O_8_Zn_7_ |
| Fw | 557.30 | 517.32 | 869.03 | 1488.95 |
| T, K | 150(2) | 100(2) | 150(2) | 100(2) |
| Crystal system | Monoclinic | Monoclinic | Triclinic | Orthorhombic |
| Space group | P2**_1_**/c | P2**_1_**/c | P-1 | P_bca_ |
| *a*, Å | 10.8214(7) | 10.6427(3) | 8.9263(3) | 13.9027(3) |
| *b*, Å | 15.4048(10) | 23.7241(5) | 19.9043(12) | 20.3855(4) |
| *c*, Å | 16.0663(11) | 10.6454(3) | 23.1393(11) | 22.9392(5) |
|  | 90 | 90 | 106.950(5) | 90 |
| *β * | 108.196(2) | 115.719(4) | 90.220(4) | 90 |
| *γ * | 90 | 90 | 94.983(4) | 90 |
| *V*, Å^3^ | 2544.3(3) | 2421.56(13) | 3915.9(3) | 6501.3(2) |
| **** | 4 | 4 | 4 | 4 |
| *ρ*_calc_, g/m^3^ | 1.455 | 1.419 | 1.474 | 1.521 |
|  mm^-1^ | 1.911 | 2.001 | 1.869 | 2.591 |
| Reflections collected | 45932 | 26343 | 38139 | 44911 |
| No. of parameters | 307 | 271 | 931 | 376 |
| Indep. reflns (*R*_int_) | 5230 (0.0455) | 5900 (0.0278) | 18223 (0.0825) | 7971 (0.0363) |
| Final R indices *R_1_^a,^ wR_2_^a^* | R1= 0.0342, wR2= 0.0663 | R1= 0.0234, wR2= 0.0527 | R1= 0.0460, wR2= 0.0472 | R1= 0.0407, wR2= 0.0740 |
| *R* indices(all data) | R1= 0.0487, wR2= 0.0737 | R1= 0.0342, wR2= 0.0538 | R1= 0.1365, wR2= 0.0556 | R1= 0.0620, wR2= 0.0780 |
| GoF *^b^* | 1.122 | 1.006 | 0.633 | 1.072 |

*^a^* *P1*  __ − _χ_  __ *P2* =  __^^ − _χ_^^^^  __^^^^^^   = 

*^b^* GoF = [Σ*w*(F_0_^2^-F_c_^2^)^2^/(*N*_rflns_ – *N*_params_)]^1/2^.

**Kinetic Studies for Polymerization of L-lactide by Complexes 1 and 2**

A J Young NMR tubecontaining *d*_8_-toluene (1 mL), THF (0.5 mL), benzyl alcohol (0.008 mmol) and L-lactide (36 mg, 31eq.) and catalyst (Zn, 0.008 mmol) was reacted at room temperature (26 ^o^C). The monomer conversion was monitored over time by ^1^H NMR spectrometry.

**Fig. 1.** Semilogarithmic plots of the L-lactide conversion stated as ln[L-lactide]_0_/[L-lactide]_t_ versus the reaction time for the polymerization of L-lactide initiated with BnOH at 26 ^o^C.
